# Effects of HCV on Basal and Tat-Induced HIV LTR Activation

**DOI:** 10.1371/journal.pone.0064956

**Published:** 2013-06-10

**Authors:** Satarupa Sengupta, Eleanor Powell, Ling Kong, Jason T. Blackard

**Affiliations:** Division of Digestive Diseases, Department of Internal Medicine, University of Cincinnati College of Medicine, Cincinnati, Ohio, United States of America; Rosalind Franklin University of Medicine and Science, United States of America

## Abstract

Hepatitis C virus (HCV) co-infection occurs in ∼30–40% of the HIV-infected population in the US. While a significant body of research suggests an adverse effect of HIV on HCV replication and disease progression, the impact of HCV on HIV infection has not been well studied. Increasing data suggest that hepatocytes and other liver cell populations can serve as reservoirs for HIV replication. Therefore, to gain insight into the impact of HCV on HIV, the effects of the HCV Core protein and infectious hepatitis C virions were evaluated on basal and Tat-induced activation of the HIV long terminal repeat (LTR) in hepatocytes. The HIV LTR was highly induced by the HIV transactivator protein Tat in hepatocytes. Activation varied according to the number of NF-kB binding sites present in the LTRs from different HIV subtypes. Involvement of the NF-kB binding pathway in LTR activation was demonstrated using an NF-kB inhibitor and deletion of the NF-kB binding sites. TNFα, a pro-inflammatory cytokine that plays an important role in HIV pathogenesis, also induced LTR activity in hepatocytes. However, HIV LTR activity was suppressed in hepatocytes in the presence of HCV Core protein, and the suppressive effect persisted in the presence of TNFα. In contrast, infectious hepatitis C virions upregulated HIV LTR activation and gene transcription. Core-mediated suppression remained unaltered in the presence of HCV NS3/4A protein, suggesting the involvement of other viral/cellular factors. These findings have significant clinical implications as they imply that HCV could accelerate HIV disease progression in HIV/HCV co-infected patients. Such analyses are important to elucidate the mechanisms by which these viruses interact and could facilitate the development of more effective therapies to treat HIV/HCV co-infection.

## Introduction

Hepatitis C virus (HCV) is the main causative agent of acute and chronic non-A, non-B hepatitis and may lead to liver cirrhosis and hepatocellular carcinoma (HCC). It is estimated that 130–170 million people worldwide are infected with HCV [1], [2]. Due to shared transmission routes, HCV co-infection is common in persons living with human immunodeficiency virus (HIV), and 30–40% of HIV -infected people in the US are co-infected with HCV [3], [4]. It is well described that HIV significantly impacts HCV infection, as HIV/HCV co-infection is associated with more rapid progression of liver disease and decreased treatment response rates compared to HCV mono-infection [5]. However, HCV may also impact HIV disease progression. For instance, HCV RNA levels are correlated with accelerated HIV disease progression [6]–[12], while HCV seropositivity is associated with an increased risk of death among treatment-experienced HIV-positive individuals [13]. Thus, additional investigation is warranted.

A key regulator of HIV gene expression is the long terminal repeat (LTR). The LTR contains binding sites for multiple cellular and virus-encoded proteins that alter LTR activity and subsequent viral gene expression. Important LTR functional elements include the transactivation response element (TAR), which binds the viral transactivator protein Tat, and the enhancer element that contains multiple NF-kB binding sites. Tat is essential for viral replication in T-lymphocytes and macrophages, while NF-kB is another potent inducible regulatory element of LTR transactivation and HIV replication. Variation exists in the number and sequence of transcription factor binding sites within the LTRs from different subtypes, and this variation influences viral pathogenesis [14]–[19].

It is well documented that CD4+ lymphocytes and macrophages are the primary sites for HIV replication, while HCV replicates primarily within hepatocytes. However, growing evidence suggests that other cell types also support replication of these viruses. For instance, extrahepatic replication of HCV has been reported in lymphocytes and monocytes/macrophages [20]–[24]. In addition, several studies also suggest that the liver can support HIV replication. For example, a CD4-independent strain of HIV that infects human hepatocytes has been isolated [25]. Similarly, Iser *et al.* observed increased HIV reverse transcriptase activity following HIV infection of hepatocyte cell lines [26]. Recently, our group demonstrated that both CXCR4- and CCR5-utilizing HIVs can infect hepatocyte cell lines, as well as primary hepatocytes [27]. Moreover, it has been shown that human hepatoma cells can transmit surface bound HIV to CD4+ T cells [28]. HIV infection of hepatic stellate cells has been reported as well [29], [30]. Apart from direct infection, HCV and/or HIV envelope proteins induce hepatic apoptosis [31]–[36]. Additionally, gp120 activates hepatic expression of interleukin 8 (IL-8), a pro-inflammatory cytokine that represents an important mediator of hepatic inflammation and antagonist of the antiviral effects of interferon (IFN) [37]–[40].

HCV consists of a positive-strand RNA genome that encodes for a single polyprotein that is cleaved by host and cellular proteases to generate at least 10 proteins. Among the four structural (Core, E1, E2, and p7) and six nonstructural (NS2, NS3, NS4A, NS4B, NS5A, and NS5B) proteins, Core is known for its gene regulatory activities. It can function as a transcriptional regulator of both cellular and viral promoters [41]–[43]. Previous reports suggest that Core exerts a stimulatory effect on the Rous Sarcoma Virus LTR and the Simian Virus 40 early promoter, while it inhibits HIV replication in lymphocyte cell lines [44]. However, data in other cell types are limited. HCV also has several immunoregulatory effects on the host that influence pathogenicity and may facilitate interactions with the HIV promoter. For example, tumor necrosis factor alpha (TNFα), a monocyte/macrophage-derived pro-inflammatory cytokine, is elevated during chronic HCV infection but also plays a pivotal role in HIV pathogenesis by inducing viral transcription via the NF-kB pathway [18], [45]–[47].

In this present study, basal and/or Tat-induced HIV LTR activation were examined in the presence of the HCV Core, TNFα, and infectious hepatitis C virions in hepatocytes.

## Materials and Methods

### Cell Culture

The human hepatic cell line Huh7.5 was cultured in Dulbecco’s modified Eagle’s medium (DMEM, GIBCO) containing 2 mM L-Glutamine and supplemented with 10% fetal calf serum (FCS), penicillin, and streptomycin (100 µg/mL). Cells were maintained at 37°C and 5% CO_2_. The HepG2 hepatocyte cell line was cultured in RPMI1640 media with 4% FCS and 4 mM L-Glutamine. The human embryonic kidney cell line 293T and hepatocytes (Huh7.5_JFH1_) constitutively expressing the infectious JFH1 strain of HCV (genotype 2a) were also cultured in complete DMEM [48], [49]. Lymphocytic Jurkat cells were maintained in complete RPMI (10% FCS, 1% penicillin/streptomycin), while the Huh7/β-gal indicator cell line was cultured in complete DMEM supplemented with 0.5 mg/mL of G418. The hepatocyte-derived HIV indicator cell line Huh7/β-gal was created by transfecting the *LacZ* gene under control of the HIV subtype B LTR into the parental Huh7 cell line (Dr. Julie AE Nelson – now at the University of North Carolina – and Tara Riddle). Stable transfectants were screened for β-galactosidase expression in the presence of the G418 selection marker.

### Plasmids

Plasmid reporter constructs containing the luciferase gene under control of the LTR (pLTR-Luc) [16] representing HIV-1 (denoted HIV in the text) subtypes A through G, a vector expressing HIV Tat (pSV2tat72) [50], the pNL4-3luc.R^−^E^−^, and pNL4-3HSA.R^−^E^−^ vectors [51], [52] were obtained from the NIH Research and Reference Reagent Program. The delNFkB construct was created by removing both NF-kB binding sites from the subtype B LTR-Luc plasmid. The pNL4-3luc.R^−^E^−^, and pNL4-3HSA.R^−^E^−^ vectors contain the firefly luciferase gene or the murine heat stable antigen (CD24) cDNA, respectively, in the *nef* gene of pNL4-3. The HCV Core (genotype 1b strain J1) expression vector – pCAGGS [53] – was a gift from Tetsuro Suzuki at the National Institute of Infectious Diseases (Japan). The HCV NS3/4A (genotype 1) gene cloned in pEF1 expression vector was a gift from Michael Gale Jr at the University of Washington University. A control vector (pCI) with no luciferase expression served as a negative control and to equalize the total amount of DNA per transfection reaction.

### Transfection, Infection, and Reagents

10,000–20,000 cells (hepatocytes and 293T cells) were seeded in 100 µL of media per well of a 96-well plate and transfected after 24 hours using the Fugene reagent (Roche, USA). 100 ng of various luciferase reporter plasmids (pLTR-Luc) were used in co-transfection experiments at a 3∶1 ratio of transfection reagent to DNA. The Jurkat cells were seeded in 24-well plate (∼2×10^6^ cells per well), and the amount of DNA for transfection was increased accordingly. The 50% tissue culture infectious dose (TCID_50_) of JFH1 virus harvested from the HCV_JFH1_ cell line was calculated as 2.68×10^6^/mL per the previously described methodology [54]. For experiments using infectious HCV, the *HCV+* and *HCV++* conditions correspond to Core protein concentrations of 3 ng/mL and 7.5 ng/mL, respectively, as measured by ELISA. Trypan blue staining was used for exclusion of non-viable cells. Pyrrolidine dithiocarbamate (PDTC; Biovision, USA) was used as a potent NF-kB inhibitor [55], and human recombinant TNFα was purchased from Prospec, USA for the experiments.

### Reporter Assay

At 48 hours post-transfection, cells were lysed for 5 minutes and extracts measured for luminescence to quantify LTR activation using the Steady-Glo luciferase assay (Promega, USA). For experiments involving the Huh7/β-gal indicator cell line, a β-gal staining assay (Sigma, USA) was performed at 48 hours post-transfection.

### FACS Analysis

HCV infected or uninfected Huh7.5 cells were transfected with the HIV expression vector pNL4-3HSA.R^−^E^−^. HIV transcription was quantified by measuring the expression of the CD24 gene inserted into the pNL4-3 *nef* gene. 48 hours post-transfection, the cells were stained with FITC-conjugated anti-CD24 antibody (clone M1/69, eBioscience, USA), fixed, washed, and analyzed with a BD Accuri C6 flow-cytometer using the program CFlow. For analysis, 10,000 live cells were gated on FSC versus SSC scatter plot, and histograms were overlapped for assessing changes in the FL1 channel (anti-CD24-FITC). The fold change was measured as the Mean Fluorescence Intensity.

### Statistical Analysis

All experiments were performed 2–3 times independently. Representative results are presented as the mean ± standard error. A two-sample *t*-test was used to evaluate statistical significance and considered statistically significant at a value of P<0.05.

## Results

### HIV LTR is Activated by Tat in Hepatocytes

**Figure 1:**
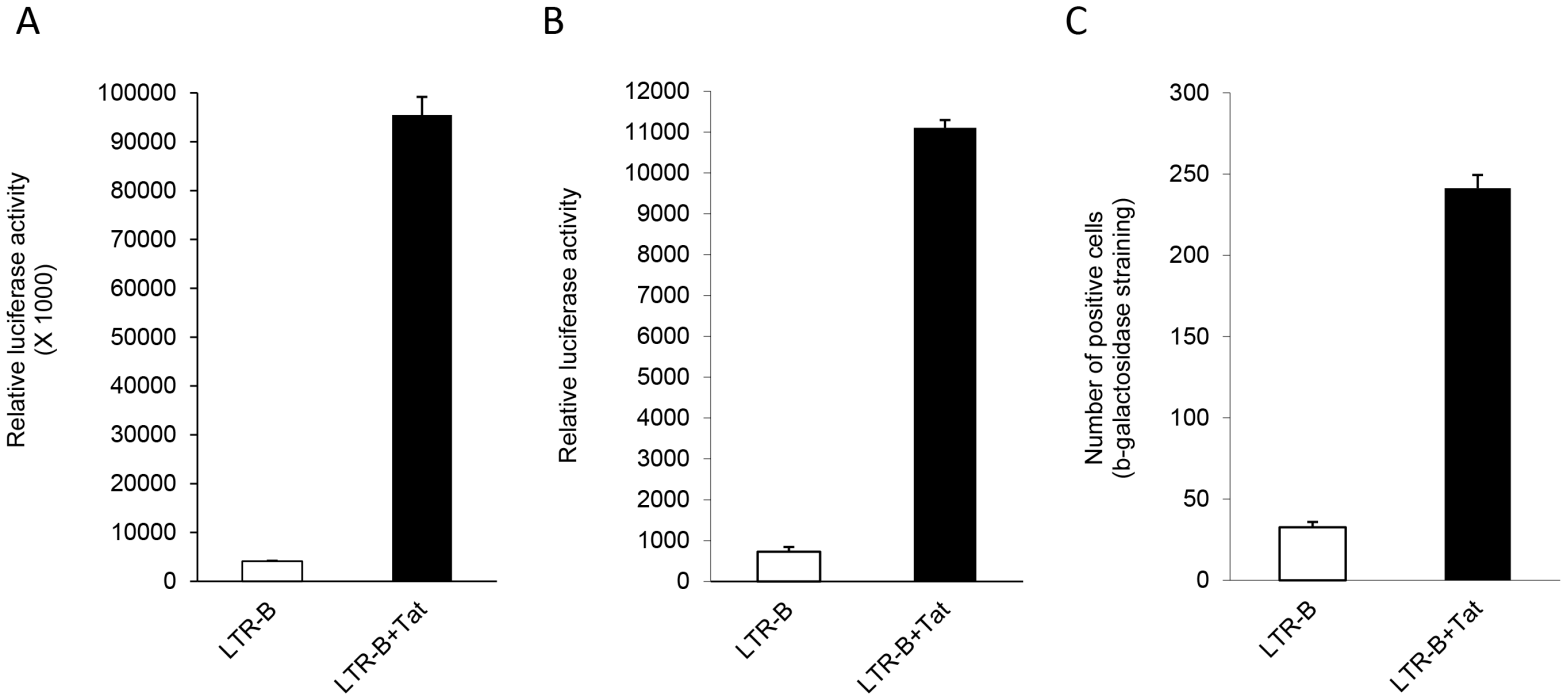
Basal and Tat-induced HIV LTR activation in Huh7.5 cells (A), HepG2 cells (B), and Huh7/β-gal cells (C). Huh7.5 and HepG2 cells were transfected with an HIV subtype B LTR (LTR-B) luciferase construct in the presence or absence of a Tat-expressing vector. 100 ng of each DNA was transfected per well of a 96-well plate. A luciferase assay was performed at 48 hours post-transfection to quantify LTR activation and was expressed as relative luciferase activity. Huh7/β-gal cells were transfected with or without a Tat expression vector. At 48 hours post-transfection, blue cells were counted after β-gal staining. White bars denote basal (no Tat) LTR activity, and black bars denote Tat-mediated LTR activation.

HIV LTR activation was elevated ∼23-fold in Huh7.5 and ∼15-fold in HepG2 cells in the presence of Tat (Figure 1A–B). Similarly, Tat-induced LTR activation in the Huh7/β-gal indicator cell line was significantly higher compared to basal levels (8-fold induction) (Figure 1C).

### HCV Core Protein Suppresses Basal and Tat-induced HIV LTR Activation in Hepatocytes

**Figure 2:**
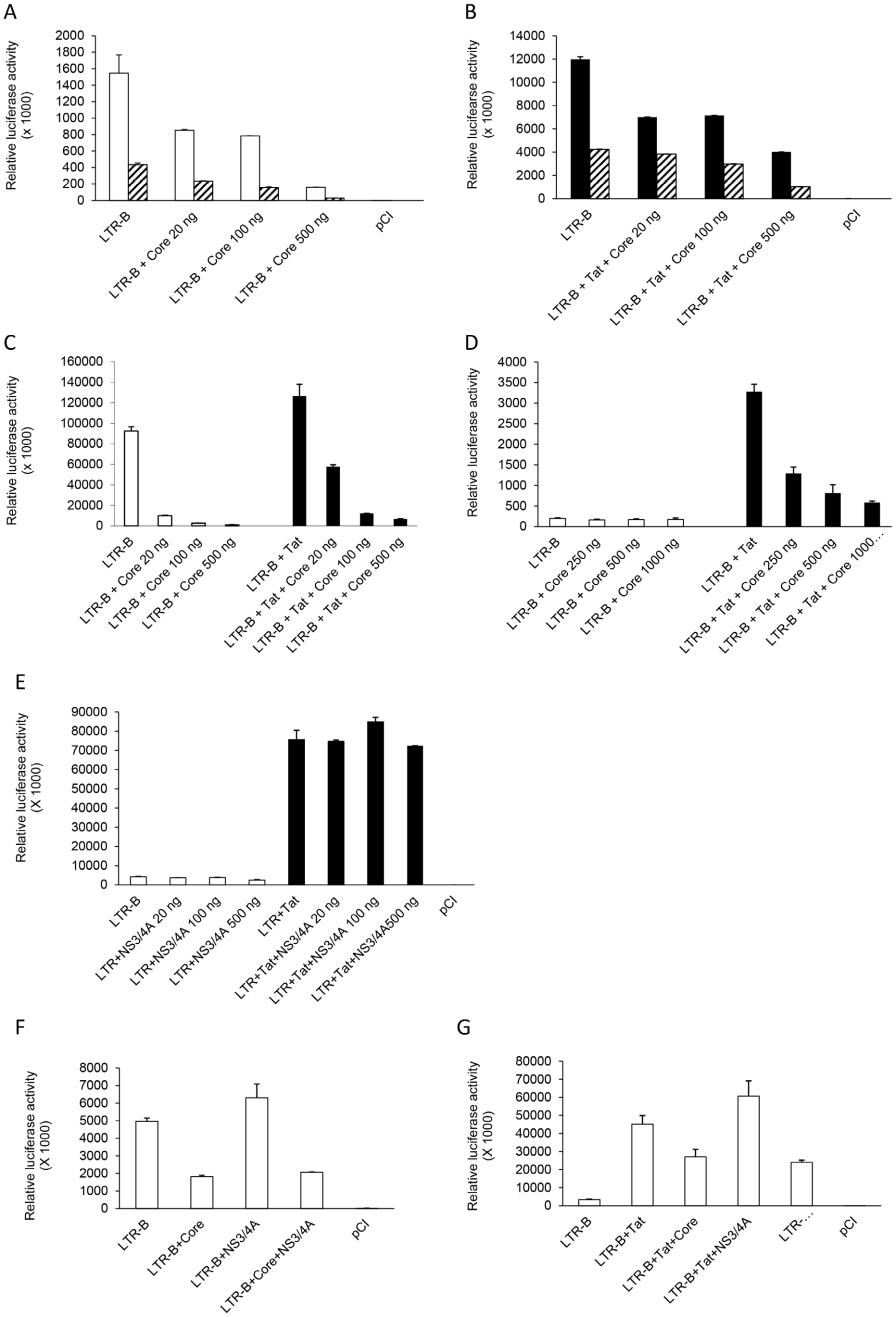
HCV Core-mediated suppression of HIV LTR activation. A dose-response experiment was performed in Huh7.5 cells for basal (**A**), as well as Tat-mediated HIV LTR activity (**B**). Huh7.5 cells (∼2×10^4^ cells per well) were seeded in a 96-well plate and co-transfected with 100 ng of HIV LTR-B luciferase construct or the delNFkB construct (hatched bars) and 20 ng, 100 ng, or 500 ng of an HCV Core expression vector with or without 100 ng of the Tat expression vector. The pCI control vector was used to equilibrate the total amount of DNA per well as well as a negative control. Luciferase assay was performed at 48 hours post-transfection and expressed as relative luciferase activity. Similar experiments were performed in 293T (**C**) and Jurkat cells (**D**). For Jurkats, ∼2×10^6^ cells were seeded per well of 24-well plate and co-transfected with 500 ng each of LTR-B or Tat and 250 ng, 500 ng, or 1000 ng for HCV Core using the transfection reagent TransIT-Jurkat (MIRUSBIO). White bars denote basal (no Tat) LTR activity, and black bars denote Tat-mediated LTR activation. A dose-response experiment with HCV NS3/4A was performed in Huh7.5 cells for basal as well as Tat-induced HIV LTR activation (**E**). The effect of HCV Core was tested in the presence or absence of HCV NS3/4A on basal (**F**) and Tat-induced LTR activation (**G**).

LTR activation was suppressed by HCV Core protein in a dose-dependent manner both in the absence (Figure 2A) and presence of Tat (Figure 2B). The percent inhibition of basal LTR activation by Core was 50%, 78%, and 95% at 20 ng, 100 ng, and 500 ng, respectively. Tat-induced LTR activation was inhibited to a lesser extent by HCV Core at lower concentrations (10% at 20 ng and 32% at 100 ng). However, at the 500 ng concentration, Core-mediated suppression was sufficient to reduce LTR activation by 91% even in the presence of Tat (Figure 2B). This suppressive effect was significantly reduced when utilizing an LTR construct with its NF-kB binding sites deleted. Additional experiments were performed in 293T cells (embryonic kidney cells) and Jurkat lymphocytes. HCV Core suppressed both basal and Tat-induced LTR activation in a dose-dependent manner in 293T cells (Figure 2C). In Jurkat lymphocytes, Core-mediated suppression on the HIV LTR was observed only in the presence of Tat (Figure 2D).

To further investigate if the Core-mediated suppressive effect on basal and Tat-induced LTR activation could be altered in the presence of another HCV protein, the effect of HCV NS3/4A protein was studied. No significant change in basal or Tat-induced LTR activation was found in the presence of NS3/4A in Huh7.5 cells (Figure 2E). Moreover, NS3/4A did not alleviate the Core-mediated suppressive effect on basal or Tat-induced LTR activation in hepatocytes (Figure 2F–G). This indicated that the suppression effect on HIV LTR was specific to the Core protein.

### TNFα Induces HIV LTR Activity in Hepatocytes

**Figure 3:**
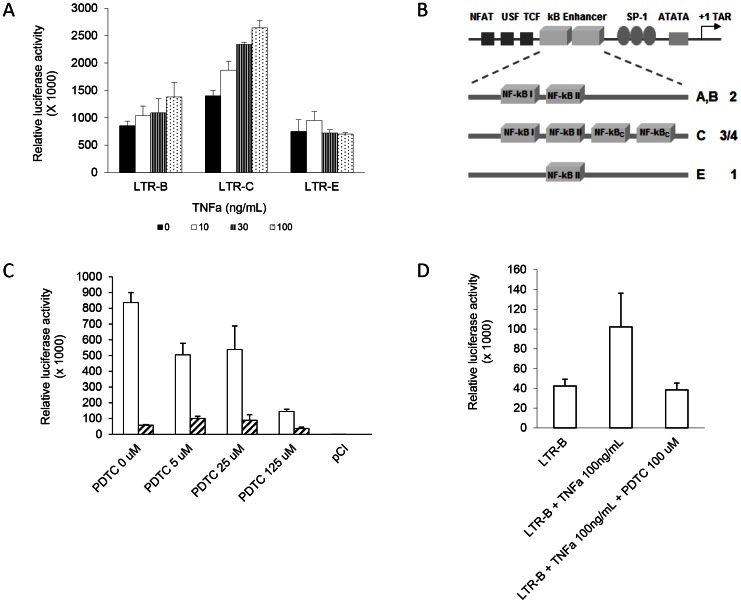
TNFα-mediated HIV LTR activation in Huh7.5 cells. Subtype B, subtype C, and subtype E LTR activation were tested in the absence or presence of increasing concentrations of TNFα (10 ng/mL, 30 ng/mL, and 100 ng/mL) (**A**). Differences in number of NF-kB binding sites according to the HIV LTR subtypes (**B**). LTR-B (or delNFkB – denoted by hatched bars) activation was detected in Huh7.5 cells in the absence or presence of the NF-kB inhibitor PDTC at concentrations of 5 µM, 25 µM, and 125 µM (**C**). TNFα-mediated (100 ng/mL) LTR activation was inhibited in the presence of the NF-kB inhibitor PDTC (100 µM) **(D)**.

Chronic HCV infection is associated with production of the pro-inflammatory cytokine TNFα *in vivo* [46], [47]. Furthermore, TNFα is an important regulatory factor in HIV pathogenesis and acts through NF-kB to activate HIV transcription [18], [56]. Therefore, the role of TNFα on HIV LTR activity was examined in hepatocytes. The LTR was activated by TNFα in a dose-dependent manner and was highest for the subtype C LTR, intermediate for the subtype B LTR, and least for the subtype E LTR (Figure 3A). This suggests that NF-kB is involved in TNFα-mediated LTR activation in hepatocytes as has been reported in lymphocytes [57], given that the subtype C LTR contains three NF-kB binding sites, while subtype B LTR contains two and subtype E has only one NF-kB binding site (Figure 3B). To further investigate the role of NF-kB in LTR activation in hepatocytes, Huh7.5 cells were treated with or without the NF-kB inhibitor (PDTC) and then transfected with HIV LTR-B. LTR activation was highest in cells with no PDTC and decreased in a dose-dependent manner in the presence of PDTC (Figure 3C). The percent inhibition of LTR activation by PDTC was 43%, 77%, and 88% at 5 µM, 25 µM, and 125 µM, respectively. No effect of PDTC was observed in Huh7.5 cells transfected with the delNFkB construct. Moreover, TNFα-induced LTR-B activation was inhibited in the presence of PDTC (Figure 3D) – but had no effect in Huh7.5 cells transfected with the delNFkB construct (data not shown) – further suggesting the involvement of NF-kB in TNFα-mediated LTR activation in hepatocytes.

### HCV Core-mediated Suppression of HIV LTR Persists in the Presence of TNFα

**Figure 4:**
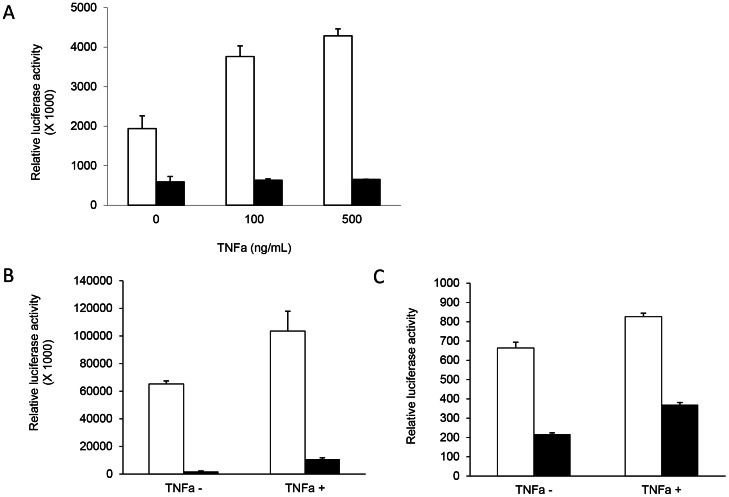
HCV Core-mediated suppression of HIV LTR activation in the presence of TNFα. LTR-B-transfected Huh7.5 cells were co-transfected with or without HCV Core and were treated with increasing concentration of TNFα (0 ng/mL, 100 ng/mL, or 500 ng/mL) (**A**). 293T (**B**) and Jurkat cells (**C**) were transfected with LTR-B and co-transfected with or without HCV Core in the presence or absence of TNFα. White bars denote the LTR-B only condition, while black bars denote the LTR-B+Core condition.

To investigate whether the suppressive effect of HCV Core protein on HIV LTR could be overcome by TNFα, Huh7.5 cells were treated with or without the recombinant TNFα and co-transfected with LTR-B in the presence or absence of HCV Core. As expected, LTR activation was higher in the cells treated with TNFα compared to untreated cells (Figure 4A). However, there was no induction by TNFα in Huh7.5 cells expressing HCV Core, and LTR activation was suppressed even at higher concentrations of TNFα (Figure 4A). Similarly, in 293T and Jurkat cells, Core-mediated suppression of the LTR persisted in the presence of TNFα (Figure 4B–C).

### Infectious HCV Upregulates HIV Transcription

**Figure 5:**
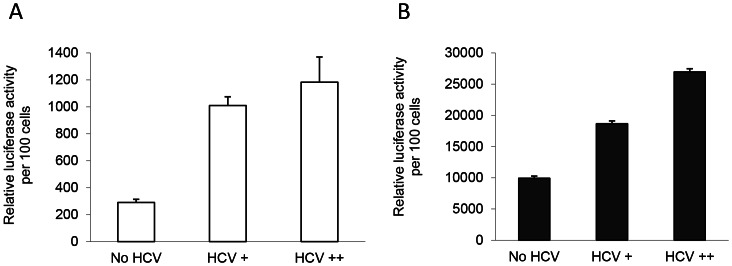
Dose-dependent increase in HIV LTR activation in HCV-infected Huh7.5 cells. The TCID_50_ of JFH1 virus harvested from the HCV_JFH1_ cell line was 2.68×10^6^/mL per using a previously described methodology [54]. The cells were infected with JFH1 virus at 3 ng/mL and 7.5 ng/mL concentrations of Core protein denoted as HCV+ and HCV++, respectively, and were transfected with HIV LTR-B in the absence (**A**) or presence of HIV Tat (**B**). White bars denote basal and black bars denote Tat-mediated LTR activation.

To investigate the effect of infectious hepatitis C virions on HIV expression in hepatocytes, Huh7.5 cells were infected with increasing amounts of infectious JFH1 virus and co-transfected with LTR-B with or without Tat. Basal LTR activity was increased 3.5–4 fold (Figure 5A), while Tat-mediated LTR activation was increased 1.8–2.7 fold (Figure 5B) compared to uninfected hepatocytes.

It has been reported that the JFH1 strain is not capable of infecting lymphocytes; however, it exhibits efficient polyprotein processing and IRES-dependent translation [58]. Thus, Jurkat cells were exposed to JFH1 virus for 4 hours, followed by transfection with LTR-B with or without Tat. In the presence of Tat, there was a slow but gradual increase in LTR-B activation (up to ∼1.4 fold) in exposed cells compared to unexposed cells. No change in basal activation of LTR-B was observed (data not shown). In addition, exposure of Jurkats to infectious HCV resulted in increased activation of the subtype C LTR at a higher level (∼1.5 fold) than the subtype B LTR (∼1.1 fold) (data not shown).

**Figure 6:**
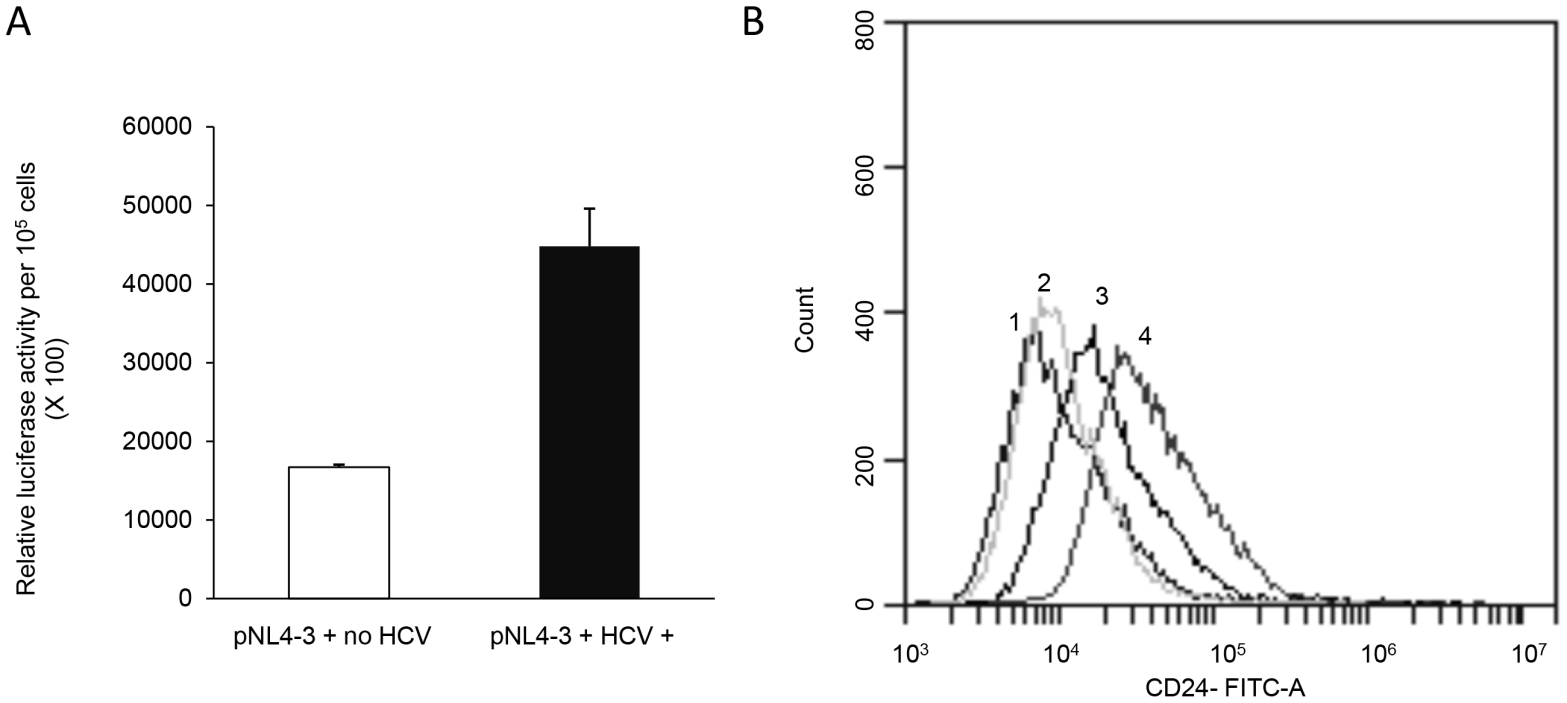
Effects of infectious HCV on HIV transcription. JFH1 virus infected or uninfected Huh7.5 cells were transfected with or without the pNL4-3luc.R^−^E^−^ vector (**A**). Increased HIV transcription (as measured by CD24 expression) in HCV-infected cells compared to HCV-uninfected cells (**B**); HCV infected or uninfected Huh7.5 cells were transfected with the HIV expression vector pNL4-3HSA.R^−^E^−^ containing the CD24 antigen as described in the methods section. The respective numbers indicate as follows: 1– HCV-uninfected Huh7.5 cells with CD24 antibody; 2– HCV-infected Huh7.5 cells with CD24 antibody; 3– HCV-uninfected Huh7.5 cells expressing pNL4-3.HSA-R^−^E^−^ with CD24 antibody; 4– HCV-infected Huh7.5 cells expressing pNL4-3.HSA-R^−^E^−^ with CD24 antibody.

To further explore the effect on HIV transcription and gene expression in hepatocytes, Huh7.5 cells were transfected with the pNL4-3luc.R^−^E^−^ vector which transcribes six HIV proteins – Gag, Pol, Vif, Tat, Rev, and Vpu. HIV transcription was inhibited by HCV Core, and the suppression effect was not altered in the presence of HCV NS3/4A (Figure S1). However, when Huh7.5 cells were infected with infectious HCV and then transfected with the pNL4-3luc.R^−^E^−^ vector, HIV transcription was increased ∼2.7 fold (Figure 6A). FACS analysis also confirmed increased HIV expression of 1.6 fold in HCV-infected Huh7.5 cells compared to HCV-uninfected Huh7.5 cells (Figure 6B). Collectively, these data indicate that infectious virions overcome Core-mediated suppression and upregulate HIV expression in hepatocytes. As HCV NS3/4A had no effect on Core-mediated suppression of HIV transcription, in the presence of infectious HCV, there are likely other viral and/or cellular factors present that alleviate Core-mediated suppression of the HIV LTR in hepatocytes.

## Discussion

While HIV can infect a variety of immune cells, such as CD4+ T lymphocytes and monocytes/macrophages, HCV is mainly hepatotropic. However, a growing number of studies demonstrate that extrahepatic replication of HCV occurs *in vivo* [20]–[23], [59]–[67]. Moreover, HIV infection of multiple liver cell populations including hepatocytes and hepatic stellate cells has been reported [25]–[28], [30]. Previous studies have reported basal LTR activation in HepG2 hepatoma cells [68], [69], although other cell types more relevant to HCV replication and HIV/HCV co-infection have not been rigorously evaluated.

In the current study, Tat-induced LTR activation was significantly elevated compared to basal activation levels in multiple hepatocyte cell lines. Furthermore, TNFα induced HIV LTR activation in hepatocytes according to the number of NF-kB binding sites. Moreover, this effect was absent when the NF-kB binding sites were deleted or when cells were incubated with an NF-kB inhibitor. The increased activation of LTR-C compared to LTR-B may also imply increased replication of subtype C HIV in liver cells, since a similar phenomenon has been reported with respect to HIV/HBV co-infection [70].

The HCV Core protein is known to regulate several viral promoters and proto-oncogenes [42], [43]. An early report also suggested that HCV Core could inhibit HIV replication [44]. Thus, it was important to explore this pathway further in other relevant cell types and in the presence or absence of other regulatory factors. Our dose-response analysis revealed that the level of inhibition of LTR activity in hepatocytes was 50% with a very low amount of Core and 95% at a higher dose. While one might expect that Tat activation would overcome the suppressive effect of HCV Core, this was not observed, as Core-mediated LTR suppression reached ∼90% at higher concentrations even in the presence of Tat. Similarly, in 293T cells and Jurkat lymphocytes, Core-mediated inhibition of Tat-induced LTR activity was pronounced (50%) even at lower doses of Core and was subsequently increased at higher doses. Moreover, HCV Core was able to overcome the inducible effect of TNFα and inhibit HIV LTR activation. This suppressive effect was specific to Core as parallel experiments showed no effect of the HCV NS3/4A protein on LTR activation (Figures 2F, 2G and S1).

The HCV Core protein regulates the NF-kB signaling pathway [71], [72]. However, this regulation is dependent upon variation within the Core protein [73], [74]. While amino acids 9–11 (RKT) of Core are responsible for the modulation of NF-kB activation, the RKP substitution fails to activate NF-kB [73]. However, the RKT sequence is well conserved in genotype 1b (Core plasmid used in this study), genotype 2a (JFH1 virus used in this study), and other HCV genotypes. Therefore, the absence of NF-kB activation due to the Core RKP substitution can be ruled out in this study, although factors other than NF-kB may be involved in this suppressive mechanism as reported previously [44]. Thus, further studies are necessary to determine whether HCV Core can directly bind to the HIV LTR or if it is involved in other protein-protein interactions that modulate the cellular transcriptional machinery and repress LTR activation.

Importantly, Figures 5 and 6 demonstrated that HIV LTR activation and gene expression were higher in HCV-infected hepatocytes compared to uninfected cells. This indicates the involvement of other essential viral factors or virus-induced cellular factors during HCV infection that overcome Core-mediated suppression and upregulate HIV activation in hepatocytes. While the mechanism of Core-mediated suppression is unknown, an earlier study in HeLaT4 cells suggested that nucleotides −65 to +3 of the LTR may be involved [44]. This indicated that the binding sites for various transcription factors including NF-kB were not involved in Core-mediated suppression. Nonetheless, upregulation of NF-kB and NF-kB-responsive genes by HCV has been reported [75]. The HCV NS5A protein is also known to be a potent transcriptional activator, and NS5A can activate NF-kB [76]–[78]. Similarly, the HCV NS3 protein can activate AP-1 and NF-kB binding and thus regulate the TNF-α promoter [79]. Moreover, one recent report suggests that the HCV NS3/4A protein upregulates HIV LTR activation and transcription [80]. A role for HIV Vpu protein (in association with HCV NS3/4A) in the stimulation of HIV transcription has also been reported [81]. However, we did not observe any impact of NS3/4A on HIV LTR activation, and addition of NS3/4A did not alter the suppressive effect of Core. Therefore, further studies involving other HCV proteins – in the presence or absence of Core – are important to characterize any positive regulatory role(s) in activating HIV LTR and overcoming Core-mediated suppression. The effects of cytokine and chemokine pathways other than TNFα might also be involved in HCV-mediated upregulation of HIV LTR and require consideration in future studies.

In summary, our finding of stimulatory effect of infectious HCV on HIV LTR activation and gene expression has important implications for HIV/HCV co-infection and implies that HCV could induce HIV activation and thus accelerate HIV disease progression in co-infected patients. In agreement, several *in vivo* studies have reported decreased CD4+ T cell counts, higher HIV RNA plasma viral loads, rapid HIV disease progression, increased mortality, and/or an increased risk of developing AIDS-defining illnesses in HIV/HCV co-infected individuals compared to HIV mono-infected patients [6], [82]–[85]. Thus, treatment of HCV infection may serve as an important strategy for reducing HIV viral load and slowing disease progression. On the other hand, it is known that HIV has deleterious effects on HCV infection. Consequently, an increase in HIV activation caused by HCV can in turn impact the clinical outcome of HCV infection and disease progression. Therefore, treatment of hepatitis C in the HIV/HCV co-infected patients could be beneficial for several reasons. Further *in vitro* and *in vivo* studies are warranted now to characterize the mechanisms by which these viruses interact with the ultimate goal of facilitating the development of more effective therapies to treat HIV/HCV co-infection.

## Supporting Information

Figure S1 HCV Core-mediated suppression of HIV transcription in the presence (black bars) or absence (white bars) of HCV NS3/4A.
(TIF)
