# Supplementary figures and images for: Effects of HCV on Basal and Tat-Induced HIV LTR Activation
Source: PLoS One. 2013 Jun 10;8(6):e64956. doi: 10.1371/journal.pone.0064956 (PMC3677892; doi:10.1371/journal.pone.0064956)

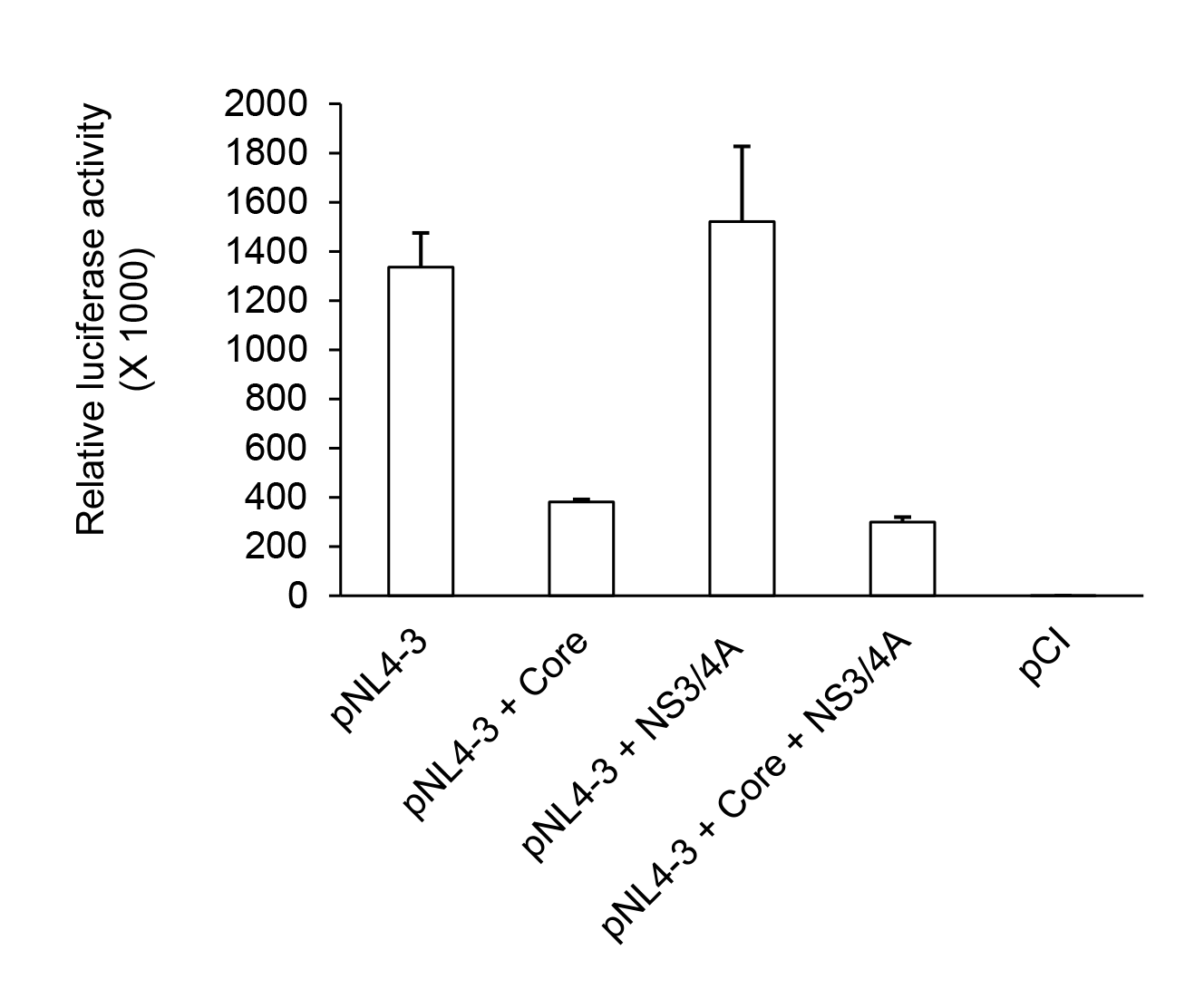

Supplement: Figure S1 — HCV Core-mediated suppression of HIV transcription in the presence (black bars) or absence (white bars) of HCV NS3/4A. (TIF) [file pone.0064956.s001.tif]
